# Leader-member exchange fosters nurses’ job and life satisfaction: The mediating effect of job crafting

**DOI:** 10.1371/journal.pone.0250789

**Published:** 2021-04-28

**Authors:** JiaLiang Pan, Chui-Yu Chiu, Kun-Shan Wu

**Affiliations:** 1 Ph.D. Program at College of Management, National Taipei University of Technology, Taipei, Taiwan, R.O.C.; 2 Department of Industrial Engineering and Management, National Taipei University of Technology, Taipei, Taiwan, R.O.C.; 3 Department of Business Administration, Tamkang University, Taipei, Taiwan, R.O.C.; University of Jyvaskyla, FINLAND

## Abstract

This study aims to evaluate the effects of leader-member exchange (LMX) on job and life satisfaction among nurses in China and to examine the mediating effect of individual and collaborative job crafting between LMX and job and life satisfaction. The study recruited 263 nurses who worked in hospitals in Zhejiang province, China. A set of self-administered questionnaires were used to measure the variables of LMX, job crafting, job and life satisfaction. The data was analyzed using the partial least square structural equation modelling (PLS-SEM). The results reveal that LMX has a significant positive influence on job crafting and job satisfaction. Collaborative job crafting has a significant positive influence on the job satisfaction of nurses, whereas individual job crafting does not. Moreover, LMX will affect job satisfaction and life satisfaction through a partial mediating effect of both individual and collaborative job crafting. Finally, the article discusses the academically and practical implications, and also provide some suggestions and directions for the future research.

## Introduction

Nurses are the key to achieving effective medical treatment [1], and are the backbone of most hospitals, and care and health facilities worldwide. Their role is critical in providing high-quality care to patients [2] and meeting their physical and emotional needs [3]. However, nurses must meet the diverse needs of patients whilst often working extended hours. Hospital leaders (nurse supervisors) often fail to make the most of nursing staff. Therefore, nurses work in an environment with scarce or insufficient resources and a lack of support related to job responsibilities (e.g., minimal assistance, insufficient human resources, and work insecurity) [4]. There is an overwhelming need for hospital supervisors to improve work conditions for nurses through job redesign.

Compared with traditional top-down and manager-centered job design, job crafting is manifested in bottom-up and employee-centered job design [5]. Researchers have explored the relationship between job crafting and outcome variables, such as job satisfaction, and found that job crafting is significantly positively related to employee job satisfaction [6–8]. However, some studies found a negative correlation between individual job crafting and job satisfaction [9], making the relationship between individual job crafting and job satisfaction unclear [10].

Since job crafting is primarily an individual activity, an employee’s perception on crafting their own job may be affected by their working environment [11]. In addition, as employees are part of an organization, their interpersonal environment may also affect job crafting [5]. Research on job crafting being affected by the employees themselves or their managers is relatively scarce. Recently, the authors found that there is a positive correlation between leadership style and job crafting [12, 13]. However, these studies focus on environmental factors and ignore the relationship between employees and leaders. Thus, this study focusses on the LMX theory [14] and evaluates the impact of LMX on job crafting.

Although, in recent years, some studies have conducted extensive research on job crafting for medical and health care employees [15–17], all the aforementioned literature focuses on the individual job crafting of participants. This may be because in certain professions it is difficult to adapt to working as an individual due to the high interdependence between departments. Collaborative job crafting of nurses, in particular, is rarely researched.

Considering the aforementioned, this study aims to: (1) study whether LMX can significantly influence nurses’ job crafting and job and life satisfaction; (2) investigate whether job crafting, both individual and collaborative, can significantly influence nurses’ job and life satisfaction; (3) explore whether job crafting has any mediating effects between LMX and nurses’ job and life satisfaction.

### Theory and hypotheses development

Based on the Job Demands-Resources (JD-R) model [18], the author referred to job crafting as “*the changes employees make to balance their job demands and job resources with their personal abilities and needs*” [19]. Tims et al. [20] demonstrated that job crafting is comprised of *“increasing structural job resources”*, *“increasing social job resources”*, *“increasing challenging job demands”*, and *“decreasing hindering job demands”*. When employees work hard to complete tasks, network with others, and reorganize their work practices, they achieve greater efficiency and better performance [5].

Some tasks, however, cannot be completed individually and require the collaboration of colleagues. For this reason, Leana et al. [9] extended the concept of job crafting to not only include individual job crafting, but also collaborative job crafting. Individual job crafting occurs when employees play an active role in changing task boundaries and shaping the way they actually work. Collaborative job crafting refers to “*employees who work together to determine how to change the task boundaries in order to fulfill shared work goals*” [9]. Leana et al. [9] proposed that individual and collaborative job crafting are different constructs, and that collaborative job crafting is positively correlated with performance.

In nursing, it is difficult to adapt to individual workloads because of the high interdependence between colleagues. Thus, this study adopts the classification of Leana et al. [9], as the concepts of individual and collaborative crafting can be used to clarify the work behaviors of nurses in the hospital.

#### The role of LMX

LMX theory is a relationship-based leadership approach, which focuses on the relationship between leaders and followers [21]. LMX helps employees gain access to substantial resources that are critical to shape their jobs [22]. Some empirical studies confirm that LMX relationship quality has a positive influence on employee job crafting [21, 23, 24], while other investigations on different cultural backgrounds have also proved that LMX has a positive impact on employee job satisfaction [23] and life satisfaction [25]. Employees with high-quality LMX reach a higher level of information exchange [26] and are supported and trusted by their leaders more, compared to employees with low-quality LMX [27]. Thus, high-quality LMX can assist employees to better understand their roles and how to create good working environments with the trust and support of leaders [21]. Based on the above literatures, the following hypotheses are proposed:

Hypothesis 1: LMX will be positively related to individual job crafting of nurses.
Hypothesis 2: LMX will be positively related to collaborative job crafting of nurses.
Hypothesis 3: LMX will be positively related to job satisfaction of nurses.
Hypothesis 4: LMX will be positively related to life satisfaction of nurses.

#### The role of job crafting

The results of some empirical studies show that job crafting has a significantly positive effect on job satisfaction [8, 28–31]. The meta-analysis of Rudolph et al. [8] confirmed that job crafting is significantly positively related to job satisfaction [6]. Job satisfaction can be improved by job crafting as people can redefine their job to meet their needs and increase the applicability, meaning, and purpose of their work [11, 19]. Recently, some scholars have indicated that individual and collaborative job crafting positively influences the job satisfaction of hotel employees and teachers [29, 32, 33]. The author indicated that job crafting has a significant positive impact on job and life satisfaction [34]. Furthermore, some studies have also confirmed a positive correlation between life satisfaction and job satisfaction [35, 36]. Based on the previous discussion, the following hypotheses are proposed:

Hypothesis 5: Individual job crafting will be positively related to individual job satisfaction of nurses.
Hypothesis 6: Collaborative job crafting will be positively related to individual job satisfaction of nurses.
Hypothesis7: Individual job crafting will be positively related to life satisfaction of nurses.
Hypothesis 8: Collaborative job crafting will be positively related to life satisfaction of nurses.
Hypothesis 9: Job satisfaction will be positively related to life satisfaction of nurses.

Other literature suggests that employees who have a good relationship with leaders are psychologically safer [37] and more motivated to receive help from leaders and offer help in return [38]. This may give employees more incentive to reinvent their duties, leading to higher job and life satisfaction. In addition to the relationships represented by direct linearity, such as Hypothesis 1–6, this study continues to investigate whether work remodeling has mediating effects. Therefore, the following hypotheses are proposed:

Hypothesis 10: Individual job crafting mediates the relationship between LMX and job satisfaction of nurses.
Hypothesis 11: Collaborative job crafting mediates the relationship between LMX and job satisfaction of nurses.
Hypothesis 12: Individual job crafting mediates the relationship between LMX and life satisfaction of nurses.
Hypothesis 13: Collaborate job crafting mediates the relationship between LMX and life satisfaction of nurses.

**Fig 1:**
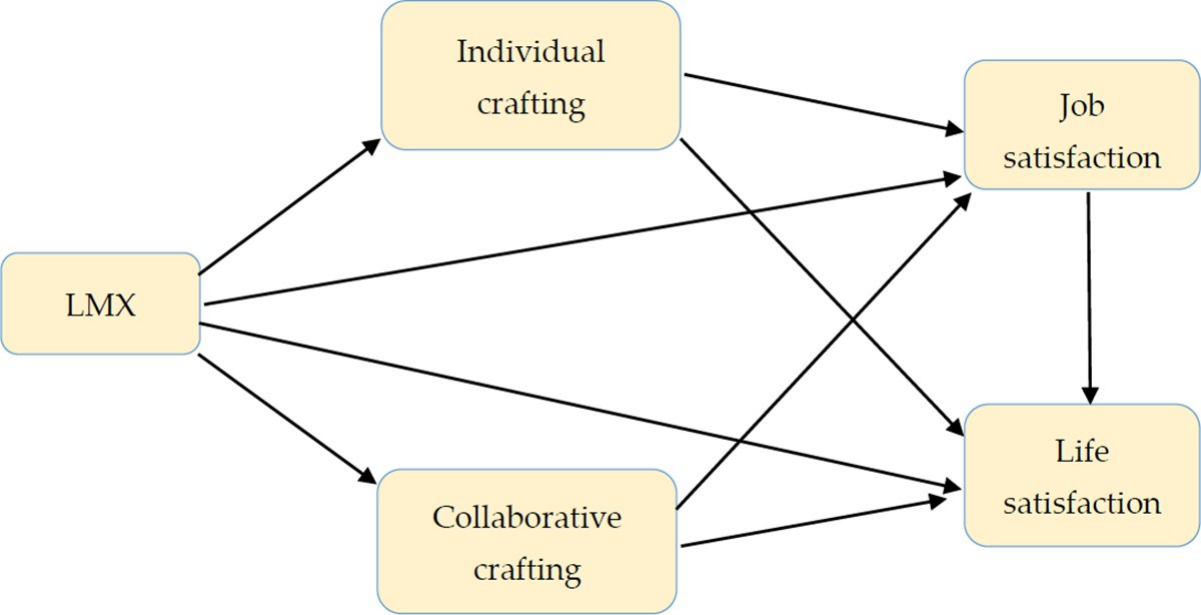
The proposed model.

According to the previous discussion, the theoretical model for this article is displayed in Fig 1.

## Method

### Ethics statement

An ethics approval was not required for this non-interventional study (e.g. surveys) as per Jiaxing University’s guidelines and relevant regulations in China. However, after hospital managers agreed to participate in this study, and ethical approval clearance and informed consent clearance were granted by the Xie, Xin Rao, Executive principle of Jiaxing University College of Medicine, hence, an ethical approach (approval) was expected. For this research, the oral consent of the participants was obtained after the principles in the Declaration of Helsinki were expressed. This study was harmless to participants as in order to preserve anonymity, names have not been used, and all data was analyzed anonymously.

### Participants and data collection

Cross-sectional survey design was employed to collect data in hospitals located within the Jiaxing City, Zhejiang province, China. The metropolis has about five tertiary hospitals. Convenient sampling was used comprising of full-time nurses over 18 years old, working at five tertiary hospitals in the Jiaxing City, Zhejiang province, China. To perform a SEM analysis, 263 samples were included, meeting Fan et al.’s [39] criteria of at least 200 samples to ensure statistical relevance.

### Measures

The questionnaire consisted of five sections: (1) demographics-this surveyed participants’ sociodemographic information, including age, education, and marital status; (2) LMX; (3) job crafting; (4) job satisfaction; and (5) life satisfaction. The original questionnaire was written in English and then translated into the participants’ native language (Chinese), as recommended by Douglas and Craig [40], to determine cultural equivalence. All major scale items were measured using a five-point Likert-type scale (1 = “strongly disagree” to 5 = “strongly agree”).

*LMX* was measured with a seven-item scale created by Janssen and Van Yperen [41]. Sample items include, “My supervisor is personally inclined to help me solve problems with my work” and “My working relationship with my supervisor is effective”. The value of Cronbach’s α for this scale was 0.921.

*Job crafting* was measured with a twelve-item scale created by Leana et al. [9]. There were six items (JC1—JC6) for individual crafting and six items (JC7—JC12) for collaborative crafting. A sample individual crafting question: do you agree that you introduce new approaches on your own to improve your work in the job? An example of a collaborative crafting question: do you agree that you work together with your coworkers to introduce new approaches to improve your work in the job? The value of Cronbach’s α for this scale was 0.933.

*Job satisfaction* was measured with the five-item scale created by Perrone et al. [42]. An example statement: on the whole, my job allows me to reach my full potential. The value of Cronbach’s α of this scale was 0.864. *Life satisfaction* was measured with the five-item scale created by Diener et al. [43]. An example statement: I am satisfied with my life. The value of Cronbach’s α of this scale was 0.915.

#### Analysis

This study used descriptive statistics, Pearson correlation, a Harman’s single-factor test and a two-step approach of partial least square structural equation modelling technique (PLS-SEM) [44] with SPSS 22.0 and VISUAL PLS 1.04b1. Descriptive statistics was used to analyze the demographic data and the mean and standard deviations of the outcome measures. The Pearson correlation coefficient was used to test the correlations among LMX, individual job crafting, collaborative job crafting, job satisfaction and life satisfaction. A Harman’s single-factor test was used to check whether there was serious concern caused from common method variance. A two-step approach of structural equation modelling (SEM) consists of confirmatory factor analysis (CFA) and SEM. CFA is to validate the measurement model, and SEM is used to test the proposed research model. PLS-SEM is presently recognized and used as the best method for multivariate analysis within social science studies [45, 46]. PLS-SEM is also frequently used in areas such as business, marketing strategy and information management to analyze data distribution, sample size and the use of formative indicators [47, 48].

## Results

### Descriptive statistics

A total of 300 questionnaires were distributed. After excluding 37 unreliable or incomplete responses, 263 questionnaires were employed for empirical analysis. The effective sample recovery rate was 87.7%. Among the respondents, the average age of the participants was 28.76 years (SD = 7.04). In total, 155 participants (58.9%) were unmarried. With regard to education, 8% had a Master’s or higher degree, 73% had a Bachelor’s degree, 19% had a college diploma or lower level of education. With respect to duration of experience in nursing (years), 20.5% had one year below, 49.8% had one to five years, 29.7% had five years above.

### Common method bias evaluation

Since the construct data considered in this study were all from the same respondents, it was difficult to avoid the problem of common method bias (CMB). As suggested by Podsakoff and Organ [49], the CMB presents some problems because one single latent construct is responsible for most of the variance. This study applied several methods to test the CMB, including Harman’s one-factor test [49]. We also performed an un-rotated principal component analysis on all of the measurement items, finding that the first factor explained only 34.91% of the total variance, which is less than the benchmark of 50% set by Podsakoff and Organ [49], so CMB was not obvious in our dataset. In addition, the construct correlation matrix (Table 2) shows that each of the inter-construct correlations was less than 0.78; however, the CMB is usually evidenced by correlations greater than 0.90 [50]. Moreover, this study also measured for the full collinearity variance inflation factors for each of the constructs, which refers to the vertical and lateral collinearity amongst the constructs [51]. These variance inflation factors can be used to evaluate the CMB and provide a more conservative test than traditional exploratory factor analysis [52]. To exclude the CMB, the variance inflation factor should be less than 3.3 [51]. In the model, the full collinearity variance inflation factors of all structures were less than 3.3. Therefore, by testing the CMB using three different methods, we can reasonably conclude that the CMB does not pose a serious threat in this study.

### Assessment of the measurement model

CFA was conducted to assess the measurement model and to test the reliability and validity of the constructs.

Firstly, the individual reliability of the items was determined by analyzing the simple loadings or correlations of the measures or indicators with their respective construct. The external loadings of the indicators must be higher than .707 in order to indicate a good fit [53]. For individual crafting, the values for item 6 (JC 6) was lower than .707. Items 2 (JS2) of the job satisfaction scale also had values lower than .707. According to Hair, Ringle, and Sarstedt [54], indicators with loadings of between .40 and .70 should only be eliminated from the scale if this leads to an increase in its composite reliability.

Secondly, the reliability of the construct was evaluated by measuring Cronbach’s alpha reliability and composite reliability. All constructs (Table 1) were found to satisfy the construct reliability requirement, having values of over .70 [55]. The empirical results implicate that reliability is satisfactory.

**Table 1:** Standardized factor loading, construct reliability and convergent validity.

Items	Standardized Factor Loading	t-value	Composite Reliability (CR)	Average Variance Extracted (AVE)	Cronbach’s α
LMX1	.738	21.643***	.937	.681	.921
LMX2	.812	28.666***			
LMX3	.847	55.422***			
LMX4	.816	26.162***			
LMX5	.894	67.388***			
LMX6	.872	56.278***			
LMX7	.789	26.187***			
JC1	.836	34.247***	.914	.680	.880
JC2	.824	27.417***			
JC3	.819	23.508***			
JC4	.816	33.481***			
JC5	.826	32.605***			
JC7	.849	47.347***	.933	.701	.909
JC8	.833	28.904***			
JC9	.870	37.046***			
JC10	.852	37.815***			
JC11	.894	56.296***			
JC12	.714	18.987***			
JS1	.836	34.983***	.917	.735	.879
JS3	.878	48.514***			
JS4	.870	31.819***			
JS5	.844	33.761***			
LS1	.875	63.323***	.937	.747	.915
LS2	.859	47.202***			
LS3	.887	48.369***			
LS4	.886	47.488***			
LS5	.813	32.270***			

Note: LMX = leader-member exchange; JC = job crafting; JS = job satisfaction; LS = life satisfaction

*** indicates *p*<0.001.

Thirdly, convergent validity was evaluated by means of the average variance extracted (AVE). The values of AVE were higher than the threshold value of 0.5 [44], which means convergent validity achieved a satisfactory level (Table 1). In addition, the cross-loading of each construct ranks first among the construct factors, and the square root of AVE for one dimension is higher than its correlation coefficient with any other dimension(s) (Table 2), in line with the criteria of Fornell and Larcker [56]. The empirical results demonstrate that the discriminant validity is satisfactory.

**Table 2:** Descriptive statistics and correlations of constructs and AVE values.

	LMX	Individual crafting	Collaborative crafting	Job satisfaction	Life satisfaction
**LMX**	**0.825**				
**Individual crafting**	0.504**	**0.824**			
**Collaborative crafting**	0.535**	0.772**	**0.837**		
**Job satisfaction**	0.626**	0.482**	0.540**	**0.857**	
**Life satisfaction**	0.514**	0.408**	0.432**	0.733**	**0.864**
**Mean**	3.676	3.821	3.742	3.316	2.795
**Standard deviation**	0.707	0.617	0.645	0.868	0.908

Note: Bold values represent the square root of average variance extracted.

### Structural model results

The hypotheses were tested applying partial least squares (PLS) method. The main advantage of PLS includes the relaxation of the normal distribution hypotheses required by the structural equation model, which enables more complex models to be easily estimated with a smaller sample size. This method focuses on the interpretation of path coefficient and variances, rather than the overall model fit [57].

Table 3 shows that LMX (beta = 0.512, p < .01) significantly affects individual crafting and explains 26.3% of the variance in individual crafting. In addition, LMX (beta = .544, p < .01) also affects collaborative crafting and explains 29.6% of the variance in collaborative crafting. Thus, Hypotheses 1 and 2 are supported.

**Table 3:** Path coefficient of structural model.

Hypothesis	Path Coefficient	Std. Error	t-value
H1: LMX→ Individual crafting	0.512**	0.058	8.823
H2: LMX→ Collaborative crafting	0.544**	0.056	9.671
H3: LMX→ Job satisfaction	0.468**	0.060	7.797
H4: LMX→ Life satisfaction	0.077	0.048	1.593
H5: Individual crafting→ Job satisfaction	0.058	0.059	0.985
H6: Collaborative crafting→ Job satisfaction	0.242**	0.080	3.041
H7: Individual crafting→ Life satisfaction	0.079	0.052	1.512
H8: Collaborative crafting→ Life satisfaction	-0.037	0.044	-0.842
H9: Job satisfaction→ Life satisfaction	0.674**	0.050	13.489

Note: ** denotes p<0.01.

Second, Table 3 shows that LMX (beta = 0.468, p < .01) significantly affects job satisfaction, whereas LMX (beta = 0.077, p>.05) did not significantly impact on life satisfaction. Consequently, the empirical results support Hypothesis 3, but not Hypothesis 4.

Third, it is found that individual crafting (beta = 0.058, p>.05) did not significantly affect job satisfaction, whereas collaborative crafting (beta = 0.242, p < .01) did significantly affect job satisfaction. Consequently, Hypothesis 5 is supported, but Hypothesis 6 is not.

Fourth, the results indicate that both individual crafting (beta = 0.079, p>.05) and collaborative crafting (beta = -0.037, p>.05) have no significant positive impact on life satisfaction, meaning Hypotheses 7 and 8 are not supported.

Lastly, the statistical results show that job satisfaction significantly positively impacts life satisfaction (beta = 0.674, p < .001) and explains 55.6% of the variance in life satisfaction. Therefore, Hypothesis 9 is supported.

**Fig 2:**
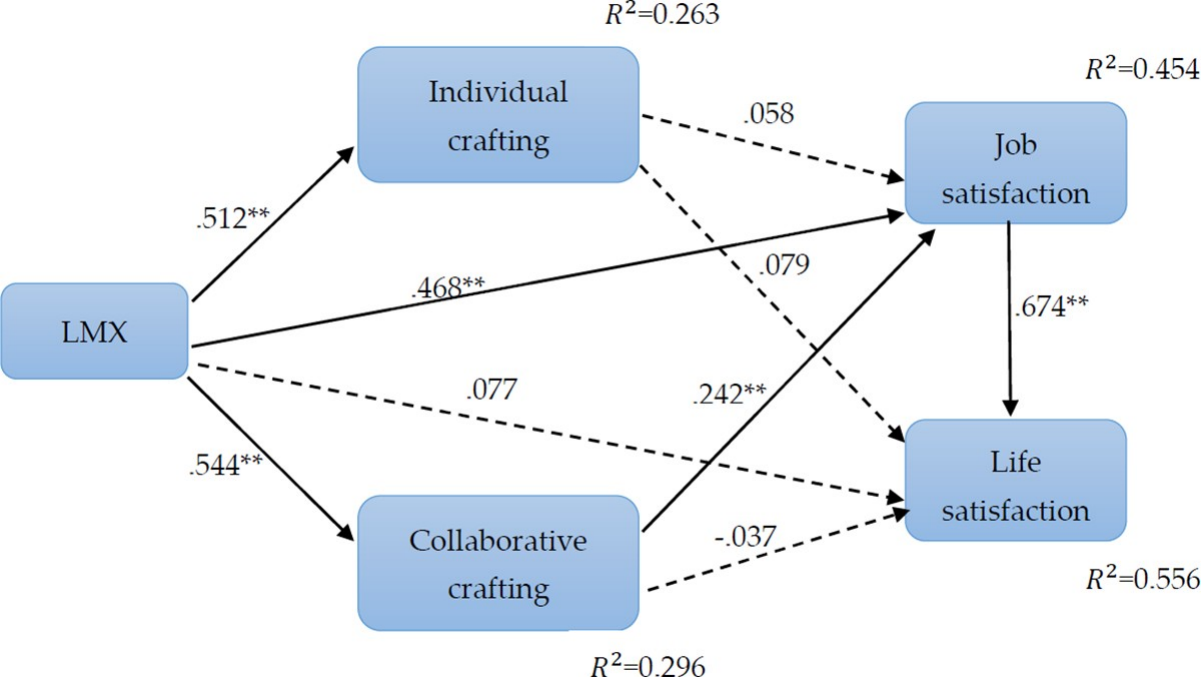
Path diagram of the structural model.

The paths of the associations among LMX, individual job crafting, collaborative job crafting, job satisfaction, and life satisfaction are shown in Fig 2.

#### Examination of mediating effects

This study also explores the mediating effect of job crafting on the relationship between LMX and job satisfaction, and the mediating effect of job crafting on the relationship between LMX and life satisfaction. First, LMX significantly positively affects both individual crafting (beta = 0.515, p<0.01) and collaborative crafting (beta = 0.546, p<0.01). Second, LMX has a significant positive influence on job satisfaction (beta = 0.631, p<0.001). Third, both individual crafting (beta = 0.169, p<0.05) and collaborative crafting (beta = 0.414, p<0.01) significantly affect job satisfaction positively. Finally, when job crafting is controlled, the impact of LMX on job satisfaction is significantly reduced, but not to zero (Table 4). Consequently, individual and collaborate job crafting have the partial mediating effect of LMX on job satisfaction. As a result, Hypotheses 10 and 11 are supported.

**Table 4:** Mediating effect of job crafting.

Independent	Mediate	Dependent	X→M(a)	X→Y(c)	X*M→Y		Outcome	Sobel test
					M→Y(b)	X→Y(c’)		
**X**	**M**	Y	Β (SE(β))	Β (SE(β))	Β (SE(β))	Β (SE(β))		Z (p-value)
LMX	JCP	JSAT	0.515**(0.0534)	0.631**(0.0481)	0.169*(0.0838)	0.466**(0.0616)	Partial mediate	1.97 (0.048)
	JCT		0.546**(0.0515)		0.414**(0.082)		Partial mediate	4.56 (0.000)
LMX	JCP	LSAT	0.515**(0.0534)	0.523**(0.0483)	0.213**(0.0842)	0.392**(0.0581)	Partial mediate	2.45 (0.014)
	JCT		0.546**(0.0515)		0.271**(0.0991)		Partial mediate	2.65 (0.008)

Note: (1) LMX = lead member exchange; JCP = individual job crafting; JCT = collaborative job crafting; JSAT = job satisfaction; LSAT = life satisfaction

(2) ** denotes p<0.01; * denotes p<0.05.

Furthermore, this study also used the Sobel test to support the mediation process suggested by Baron and Kenny [58]. Table 4 confirms that both individual job crafting (t-value = 1.97, p<0.05) and collaborative job crafting (t-value = 4.56, p<0.01) have partially mediating effects on the association between LMX and job satisfaction. Similarly, it also confirms that individual job crafting (t-value = 2.45, p<0.05) and collaborative job crafting (t-value = 2.65, p<0.01) have partially mediating effects on the association between LMX and life satisfaction. As a result, Hypotheses 12 and 13 are supported.

## Discussion

This study examined the relationships between LMX and job satisfaction and life satisfaction in the nurse industry, specifically considering the mediating role of job crafting. We have tested the convergent and discriminant validity by confirmatory factor analysis. The findings are well in support of defining the various unique dimensions of used constructs. The major findings are as follows.

First, the results indicate that LMX is a significant antecedent for both individual and collaborative job crafting. That is, the results show that high quality exchange relationships facilitate to develop nurses’ job crafting behavior. This result is consistent to what was found by Leana et al. [9] about the positive role that a supportive supervision plays in encouraging job crating. This finding is also a response to Lichtenthaler and Fischbach’s [59] call for research on the leader’s role in job crafting. This finding concurs with the propositions of several studies [21, 23, 24].

Second, in conformity with previous study [23], we put forward evidence to support the significant influence of LMX and job satisfaction of nurses in China. That is to say, in Chinese culture, “in-group” employees tend to have higher job satisfaction. However, the results reveal that LMX does not significantly influence the life satisfaction of nurses in China, which does not concur with the propositions of [25, 60]. The discrepancy is possibly caused by the inclusion of individual and collaborative job crafting as an intervening variable in this research, which may weaken the direct effect of LMX on life satisfaction.

Third, the results obtained reveal the inexistence of a direct relationship between individual job crafting and job satisfaction, whereas the collaborative crafting had a direct relationship. Collaborate job crafting enhances job resources. When nurses have adequate resources in their hospitals, they are more likely to experience satisfaction and overcome challenging situations. Therefore, nurses with greater collaborate job crafting can increase job satisfaction. Villajos et al. [61] also found that not all dimensions of job crafting predicted job satisfaction. Llorente-Alonso and Topa [62] found that the inexistence of a direct relationship between either type of job crafting and job satisfaction. Leana et al. [9] found that, contrary to their hypothesis, individual job crafting was negative associated with job satisfaction, although collaborative job crafting was found to predict higher scores for this variable.

Fourth, job crafting, both individual and collaborative, does not significantly influence the life satisfaction of nurses, diverging from the results of Rotzinger [35]. The reason for this discrepancy may be because, for nursing practitioners, long-term exposure to high-pressure work environments coupled with irregular work schedules (shifts) has no significant positive impact on life satisfaction, regardless of individual job crafting or collaborative job crafting.

Finally, the findings highlight the mediating effect of individual and collaborate job crafting on the relationship between LMX and job satisfaction, between LMX and life satisfaction. Better quality of relationship between leader (supervisor) and member (employee) leads to job crafting and hence better job satisfaction and life satisfaction. Moreover, the mediating effect of collaborative job crafting is greater than that of individual job crafting. This finding suggests that managers need to focus on leadership style in order to develop nurses’ job and life satisfaction through building collaborative job crafting.

### Theoretical and practical implications

The theoretical contributions of this research are threefold. First, drawing on social exchange theory [63] and the LMX theory [64], this study explored how LMX is associated with job crafting. This research provided the evidence of the significant influence of LMX on individual and collaborate job crafting in the workplace. Higher quality exchange relationships can facilitate to develop nurses’ job crafting behavior. Second, this study contributes to the development of job crafting literature by quantifying the relationship between job crafting and job satisfaction and life satisfaction within the context of the nurse industry. The findings reported by this study contribute new evidence in support of the theory that postulates the existence of two different types of job crafting (individual and collaborative job crafting). Third, the current research findings contribute to the extant knowledge base by uncovering the role of job crafting in the link between LMX and nurse job satisfaction and life satisfaction. Thus, this research proposes that job crafting may be seen as an important checkpoint in detecting how LMX influences job satisfaction and life satisfaction.

In terms of practical implications, the study found that LMX plays a significant role in job crafting and job and life satisfaction. Therefore, it is suggested that the relevant medical institutions should pay more attention to their nurses and/or give them the authority to reasonably adjust and improve their own workflow, according to their needs and the needs of the medical institution, to improve their job crafting. It is also recommended that hospitals provide nursing staff with more specialized instruction, such as tutorial systems to faster familiarize nurses with the duties and related skills required in their roles, in order to enhance their job satisfaction. This study also suggests that hospital supervisors establish suitable work adjustment mechanisms (e.g. reduced working hours) and welfare mechanisms, and make use of on-site education and training to strengthen the autonomous learning and team cooperation ability, and raise job satisfaction, to further strengthen nurses’ quality of life and thus improving life satisfaction.

### Limitations and directions for future research

This article has some limitations. First, the survey was only conducted in one province in China, so the results may not be generalized to other hospital’s nurses. In the future, to provide more insight into this issue, as well as the differences between countries/regions or hospital level, future studies should integrate data from different countries/regions or hospital level to make the results more generalizable.

Second, the data used was collected through convenient sampling. Although this form of data collection has often been used in former studies, the approach limits the extent of possible generalization. Future studies should endeavor to use other sampling methods to clarify the distribution of population characteristics.

## Conclusion

Although some studies have presented that LMX is an important antecedent of employees’ job and life satisfaction, there is little research on whether job crafting can mediate the association between LMX and nurses’ job and life satisfaction. This study aimed to explore whether LMX fosters job and life satisfaction among nurses in China through the mediating effect of job crafting. The empirical results demonstrate that the influence of LMX on job satisfaction and life satisfaction is partly mediated by individual and collaborative job crafting; however, collaborative job crafting has a greater mediating effect than individual job crafting.
